# Post-spin Stretch Improves Mechanical Properties, Reduces Necking, and Reverts Effects of Aging in Biomimetic Artificial Spider Silk Fibers

**DOI:** 10.1021/acsapm.4c02192

**Published:** 2024-11-20

**Authors:** Gabriele Greco, Benjamin Schmuck, Fredrik G. Bäcklund, Günter Reiter, Anna Rising

**Affiliations:** † Department of Animal Biosciences, Swedish University of Agricultural Sciences, Box 7011, Uppsala 750 07, Sweden; ‡ Department of Medicine Huddinge, Karolinska Institutet, Neo, Huddinge 141 83, Sweden; § Division Materials and Production, Department of Polymers, Fibers and Composites, RISE Research Institutes of Sweden, Mölndal 431 53, Sweden; ∥ Physikalisches Institut, Albert-Ludwigs-Universität Freiburg, Hermann-Herder-Straße 3, Freiburg 79104, Germany

**Keywords:** wet-spinning, protein fibers, biobased fibers, polymeric fibers, polymeric materials

## Abstract

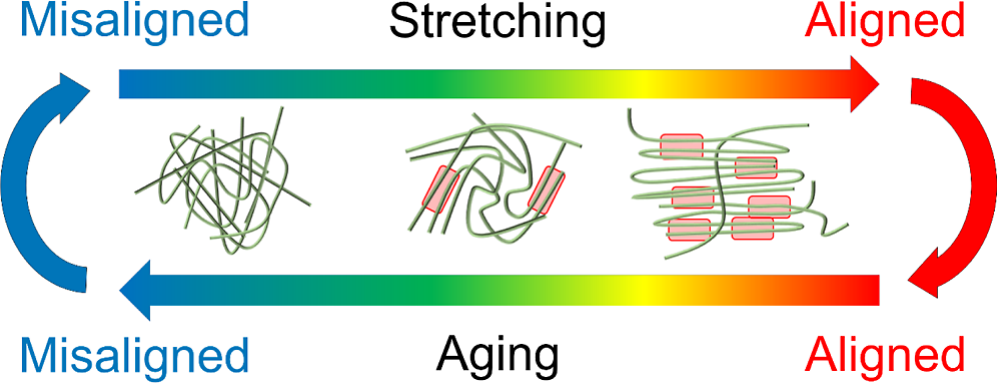

Recent biotechnological advancements in protein production and development of biomimetic spinning procedures make artificial spider silk a promising alternative to petroleum-based fibers. To enhance the competitiveness of artificial silk in terms of mechanical properties, refining the spinning techniques is imperative. One potential strategy involves the integration of post-spin stretching, known to improve fiber strength and stiffness while potentially offering additional advantages. Here, we demonstrate that post-spin stretching not only enhances the mechanical properties of artificial silk fibers but also restores a higher and more uniform alignment of the protein chains, leading to a higher fiber toughness. Additionally, fiber properties may be reduced by processes, such as aging, that cause increased network entropy. Post-spin stretching was found to partially restore the initial properties of fibers exposed aging. Finally, we propose to use the degree of necking as a simple measure of fiber quality in the development of spinning procedures for biobased fibers.

## Introduction

The negative environmental impact of synthetic plastic-based fibers underscores the urgent need for innovative, eco-friendly materials with high mechanical performance for diverse applications, such as the textile industry.^1^ In this context, biomimetic artificial silk fibers obtained from the recombinant spider silk protein NT2RepCT are promising since they are produced under environmentally friendly conditions.^2^ Moreover, recent technological achievements made it possible to produce these proteins and spin artificial silk fibers with scalable methods that are commonly used in industrial processes.^3,4^ However, the protocols for spinning fibers, including artificial spider silk, are usually dependent on fine-tuning several parameters to obtain a fiber with optimized mechanical properties.^5^ In a recent report, we explored the influence of 93 different spinning conditions on the mechanical properties of the resulting fibers, suggesting that the application of a post-spin stretch was the factor with the greatest impact on tensile strength.^6^

**Figure 1:**
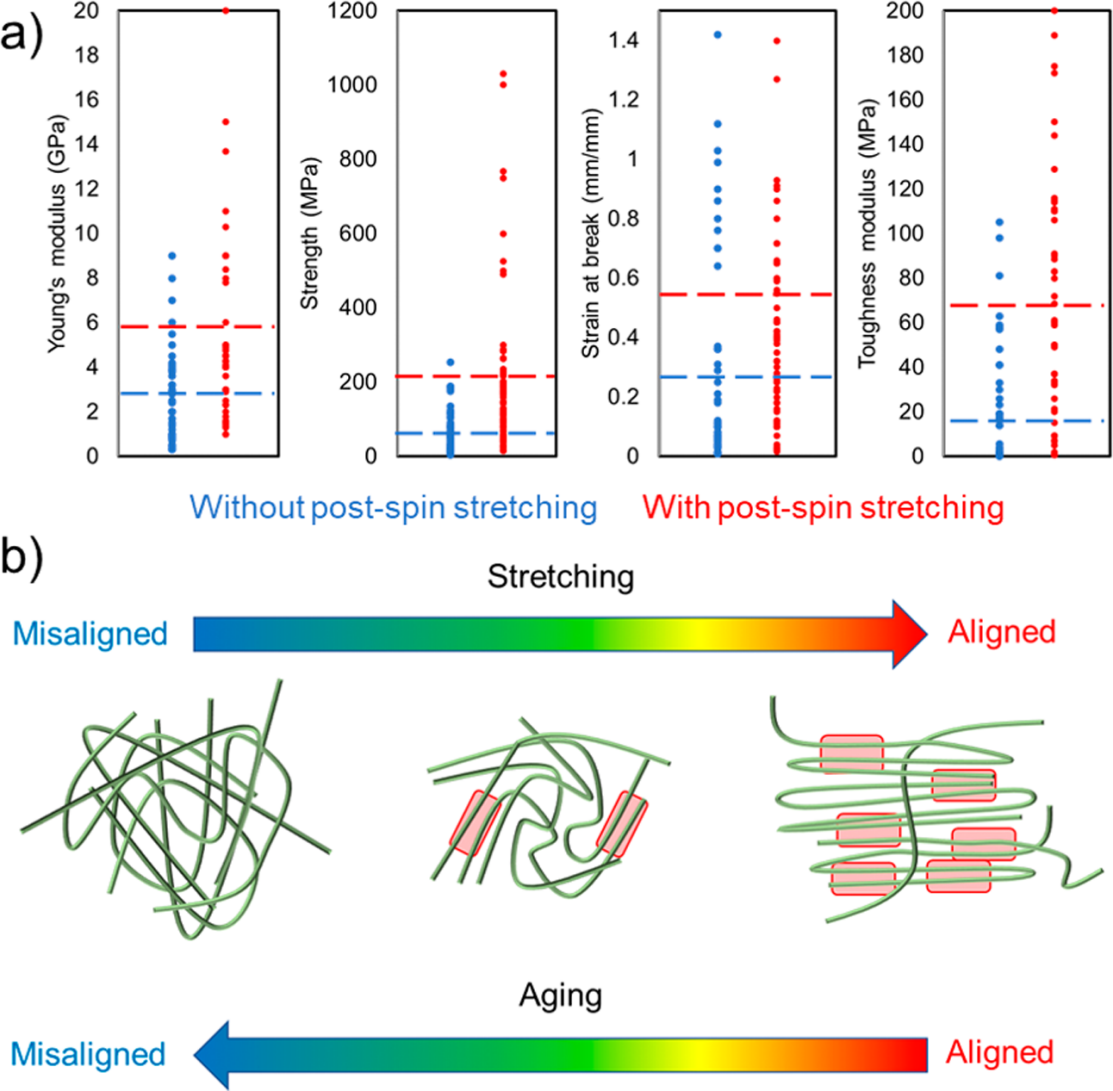
(a) Scatter plot of the mean values of Young’s modulus, strength, strain at break, and toughness modulus of fibers produced from regenerated *Bombyx mori* silk and recombinant spider silk proteins. The average of these mechanical properties (represented by dashed lines) are in general higher for fibers that were subjected to post-spin stretch. The data was obtained from refs (3, 4, 14, 16–19, 28, 52, 55–80) (b) Schematic illustration of the effects of post-spin stretching and aging of a network of polymer chains within a fiber. Aging tends to increase the state of disorder within the fiber, whereas post-spin stretching tends to increase the level of molecular order. Highlighted red regions indicate higher intermolecular interaction.

Post-spin stretching (PSS) is a general method to improve the mechanical properties of polymeric fibers, artificial silk included^7,8^ (Figure 1a). This procedure involves controlled deformation of the spun fibers to a certain strain level, thereby inducing a higher orientation of the polymer chains in the network^9,10^ (Figure 1b). For polymeric fibers and also for silk, it is known that a high degree of orientation and alignment of molecular chains is beneficial for enhancing intermolecular interactions and improving mechanical properties, e.g., strength and Young’s modulus of fibers.^10−15^

The application of PSS to silk fibers can be done during or immediately after fiber spinning, either in the presence or absence of different solvents. Consequently, the effects of PSS on fiber mechanical properties could differ according to the specific protocol used.^5,7,16−19^ For this reason, understanding how post-spin stretching affects the mechanical properties of artificial silk fibers is of high interest.

While post-spin stretching may improve the mechanical properties of artificial silk fibers, on the contrary, aging may have a deteriorating effect due to an increased molecular disorder in the polypeptide network and structural heterogeneity.^54^ In particular, aging induces a structural reorganization within the fibers toward a more stable thermodynamic state.^20^ In principle, the high level of disorder of polypeptide chains established in aged fibers can be reversed by applying a strain, i.e., stretching the fiber. If this applies also to silk fibers is not known.

The mechanical properties of polymeric fibers are not solely defined by their numerical values but also by the shape of their stress–strain curves. This is because the shape of these curves directly reflects the internal structure of the polymer’s chain network.^21−23^ For instance, in polymeric materials, the degree of necking observed in engineering stress–strain curves can signal the presence of structural or morphological heterogeneities, as well as mechanical defects within the fibers.^9,24−26^ Despite the fact that necking is frequently seen in wet-spun fibers and could be a strong indication of their quality, it is often underexplored and overlooked in research.^16,18,19,27−33,35^ In this study, we re-evaluate the data reported in Schmuck et al.^6^ and compare it to PSS performed using different conditions on freshly spun and aged wet-spun artificial silk fibers. In particular, we show that PSS improved the strength and Young’s modulus of this material, which is known to be a direct consequence of the higher overall level of molecular order. Furthermore, PSS can also be used to reduce structural heterogeneities, which can act as defects, in the fiber. To assess the presence of structural heterogeneities quantitatively, we determined the degree of necking from the engineering stress–strain curves since it reflects morphological and structural heterogeneities. Finally, we show that PSS represents a powerful approach for reverting the deteriorating effects that aging has on artificial silk fibers.

## Results and Discussion

**Figure 2:**
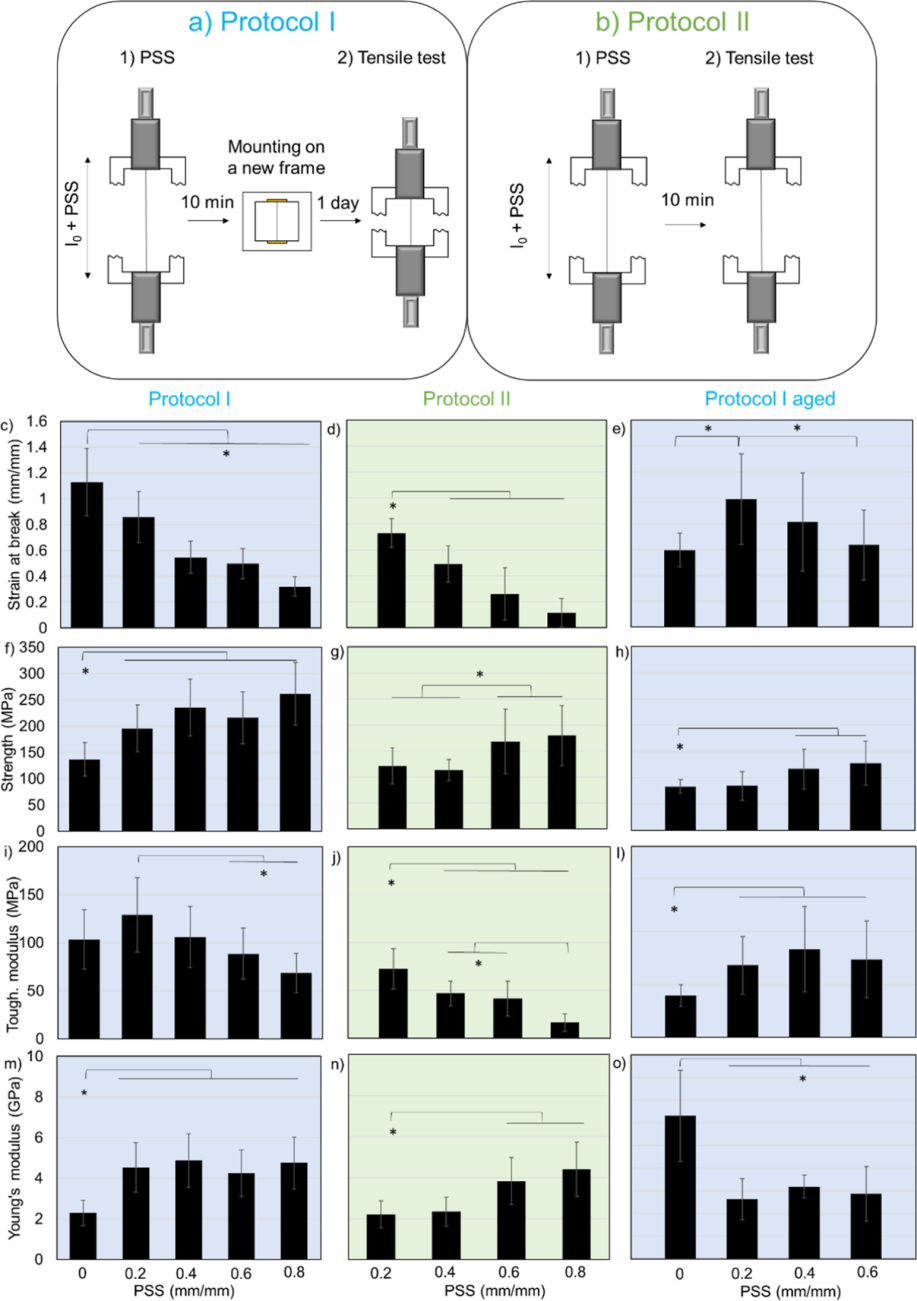
Different experimental protocols employed for applying post-spin stretching (PSS). (a) Protocol I consisted of applying a PSS to a fiber, allowing it to rest for 10 min, followed by removing it from the tensile tester. This fiber was then remounted on a new paper frame and subjected to a tensile test after 1 day. This data is shown in panels (c, f, i, and m) and were obtained from Schmuck et al.^6^ Protocol I was also employed for fibers that were aged for three months in a humid environment. (b) Protocol II consisted of applying a PSS to a fiber, allowing it to rest for 10 min, followed immediately by a tensile test without removing the fiber from the machine. (c–o) Mechanical properties of the fibers for different maximum strain levels of PSS and for different protocols employed for applying PSS. Stars indicate that the difference is significant with *p*-value <0.05 and the error bars are the standard deviations.

Artificial silk fibers were collected from the spinning bath in an automated process and dried on plastic frames as described previously.^6^ To assess the effects of PSS, we reevaluated data reported in Schmuck et al.,^6^ where the fibers were stretched in air to different levels of strain (0.2, 0.4, 0.6, and 0.8), and compared them with results from another mode of stretching. Protocol I was used to obtain the PSS data described in Schmuck et al.^6^ (Figure 2a), where the fibers were allowed to rest for 10 min after PSS to ensure that residual stresses were minimal (Figure S1), before being removed from the machine and mounted on a new paper frame. After 1 day, these fibers were subjected to a tensile test. Protocol I was also used to generate new data, for the purpose of studying artificial silk fibers that were aged for three months while being restrained on the frames where they were originally collected. Since fibers exposed to these conditions usually fractured at a stretching factor of 0.6, we could only apply PSS up to this strain level. In protocol II (Figure 2b), the fibers were subjected to PSS and allowed to rest for 10 min, followed by a tensile test without removing the fiber from the machine at any point. Thus, for Protocol II the fibers were not relaxed to the same extent compared to Protocol I.

**Figure 3:**
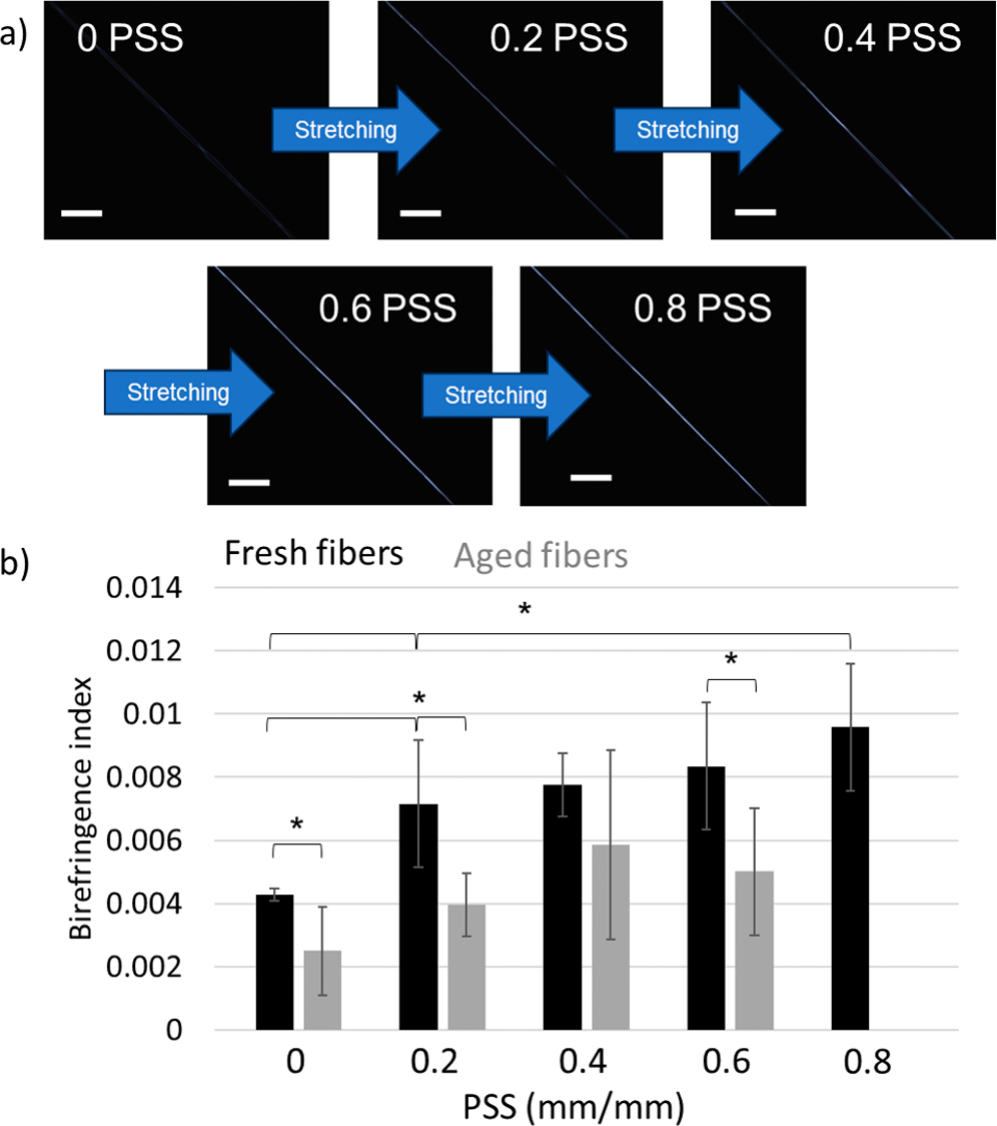
(a) Representative polarized light images (sample between crossed polarizers) of a NT2RepCT fiber at different levels of PSS. From these images, it is possible to see that PSS increases the birefringence of fiber. Before PSS (PSS = 0 means as spun fibers), the birefringence intensity is rather low and not uniform along the fiber demonstrating heterogeneities in the orientation of polymer chains. Scale bars are 100 μm. (b) Birefringence index measured for fresh and aged fibers at different level of PSS (Protocol I). PSS of 0.8 mm/mm could not be applied to aged fibers because they broke at lower levels of strain. Stars indicate that the difference is significant with *p*-value <0.05 and the error bars are the standard deviations.

The effect of both Protocol I and II is that the strain at break of fresh fibers subjected to PSS decreased (Figure 2c,d), consistent with observations for other polymeric materials.^14,35^ The strain at break of the aged fibers was significantly lower compared to fresh control fibers (Figure 2e). Interestingly, applying a small post-spin stretch factor (0.2) to aged fibers significantly increased the strain at break. The improvement in mechanical properties obtained by PSS suggests that protein degradation is not the main contributing factor to the age related effect. Instead, we speculate that an heterogeneous organization of the proteins may lead to premature fracture due to suboptimal load dissipation,^9,36^ and that stretching leads to a more uniform organization. This is supported by the enhanced brightness and uniformity of birefringence, and increased birefringence index detected by polarized light microscopy (Figure 3). Specifically, the birefringence index of aged fibers was lower than that of fresh fibers (Figure 3b), but when subjected to a 0.2 PSS the index was restored, indicating that the initial properties of fresh fibers can be partially restored in aged fibers by means of PSS. Stretching beyond the 0.2 PSS level, did not improve the strain at break further but resulted in a small increase in strength (Figure 2e,h).

The strength of the fresh fibers subjected to PSS in air increased, consistent with many observations for silk and synthetic polymer fibers^10−15^ (Figure 2f–g). This increase in mechanical strength can be ascribed to the higher degree of orientation of the polypeptide chains after PSS (Figure 3b), which is commonly observed for polymeric materials including silk.^9,10,15^ As expected, we observed also a decrease in fiber diameter of the stretched fibers (Figure S2).^6,15,28,37^ The fibers that were subjected to PSS with Protocol II displayed lower strength than those that were allowed to relax after PSS (Protocol I). This difference could potentially be explained by the assumption that stretched polymers, when allowed to relax, can partially restore interactions (i.e., self-healing) that were lost during stretching.^38−40^ The strength of the fibers that were aged for 3 months was significantly lower than that of fresh fibers reported in our previous work.^6^ As for other polymeric materials, this is probably due to that aging induced increased conformational disorder, which leads to suboptimal load distribution capacity of the protein network.^20,41^

The modulus of toughness describes how much energy a material can absorb before rupturing and is defined by the area under the stress–strain curve. Here, the experimentally observed variations of strength and strain at break as a function of the strain applied during PSS resulted in the occurrence of an optimum (maximum) toughness modulus at a 0.2 strain applied during PSS with Protocol I (Figure 2i). An optimum was observed also for aged fibers subjected to PSS, and in this case, the optimum was at a strain level of 0.4. This is not the case for the fibers that were PSS using Protocol II (Figure 2j) which displayed a constant decrease in toughness modulus with the level of strain applied with PSS.

**Figure 4:**
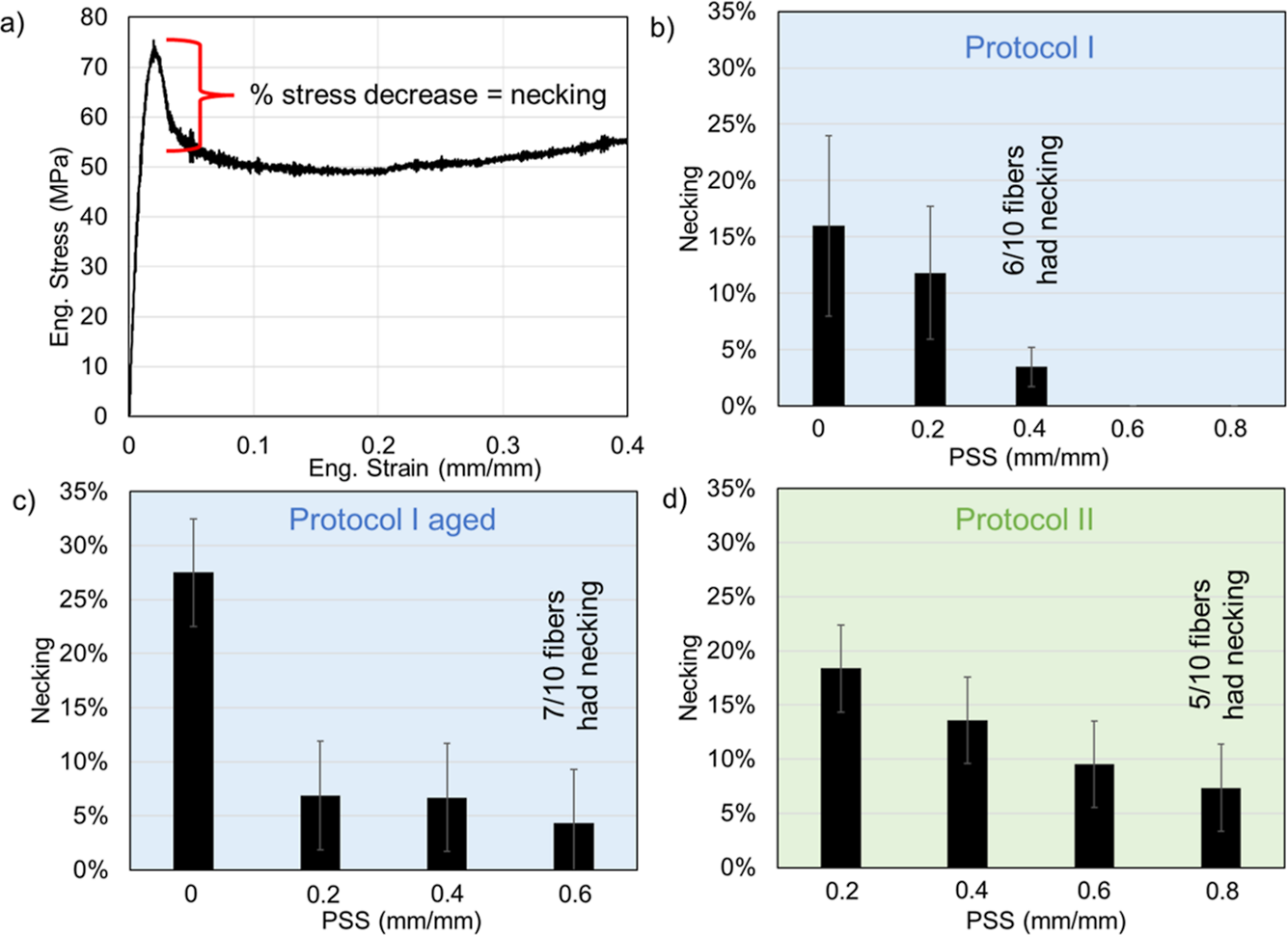
(a) Illustration showing how necking is quantified (degree of necking = “decrease in stress from yield stress”/“yield stress”). Necking values of the fibers at different levels of PSS for (b) protocol I, (c) protocol I on aged fibers, and (d) protocol II. The error bars are the standard deviations.

The Young’s modulus of the fresh fibers subjected to PSS with both Protocol I and II increased with the level of stretching (Figure 2m,n). Again, this is in agreement with what has been observed for many polymeric materials and can be explained by the higher orientation of the polymer chains in the network induced by PSS.^9,10,14,35^ The fibers that were aged displayed a much higher Young’s modulus compared to the freshly spun fibers (Figure 2o). The observed improvement in Young’s modulus serves as another indication that protein degradation does not play a major role during fiber aging for three months. Instead the increased Young’s modulus can be explained by the assumption that protein chain relaxation occurring in the course of aging results in increased disorder in the network, which increases the number of topological constraints among the protein chains.^20,42^ When a strain is applied to the chain network, the topological interaction of the chains leads to a local stress concentration, which could make the initial mechanical response of the material stiffer.^21,43−45^ This also agrees with the general observation that aging makes most polymer materials stiffer, including native spider silk.^46−48^ In this context, when a PSS is applied to the aged fiber, disordered regions probably become more ordered, which partially restore the initial level of order and thus the fiber became less stiff (Young’s modulus was decreased from 6.3 to 2.6 GPa, Figure 4o).

The mechanical qualities of fibers are defined by the strain at break, strength, Young’s modulus, and toughness modulus. Furthermore, the shape and characteristic features of the associated stress–strain curves can reflect structural organization and changes induced by stretching.^21−23^ Of particular interest is necking, which is a phenomenon that can be observed as a reduction in stress after the yield point in the engineering stress–strain graphs (Figures 4a and S3–S5). Necking is a consequence of plastic instability and a nonuniform deformation of the material,^9,49,50^ and in polymers, the phenomenon indicates the presence of regions of mechanical weakness or heterogeneous structures at all scales. Thus, fibers that do not display necking are in principle more uniform with respect to fibers that display necking. For polymeric fibers, artificial silk included, necking is commonly observed but seldom discussed in a quantitative and detailed way.^16,18,19,27−34^ We observed that for the artificial spider silk fibers, the degree of necking consistently decreased when PSS was applied, regardless of the protocol used (Figure 4b–d). This is in agreement with previously published qualitative observations.^35,37,51^ In particular, for fresh fibers subjected to Protocol I at strains of 0.6 and 0.8, no necking was observed (Figure 4b). At a PSS strain of 0.4, 40% of the fibers did not show necking while 60% of the investigated fibers showed a minor degree of necking. Interestingly, the amount of necking was on average higher for fibers undergoing protocol II (Figure 4d), again probably due to insufficient relaxation (compared to Protocol I) after PSS.^35^ Notably, for aged fibers (Figure 4c), necking was highest, confirming the notion that aging increases the molecular disorder. By applying post-spin stretching to aged fibers, we were able to significantly reduce the level of necking from 27% to approximately 6%. However, even at a PSS level of 0.6, we observed that 70% of the aged fibers still exhibited necking, whereas necking was absent in fresh fibers subjected to the same level of PSS.

**Figure 5:**
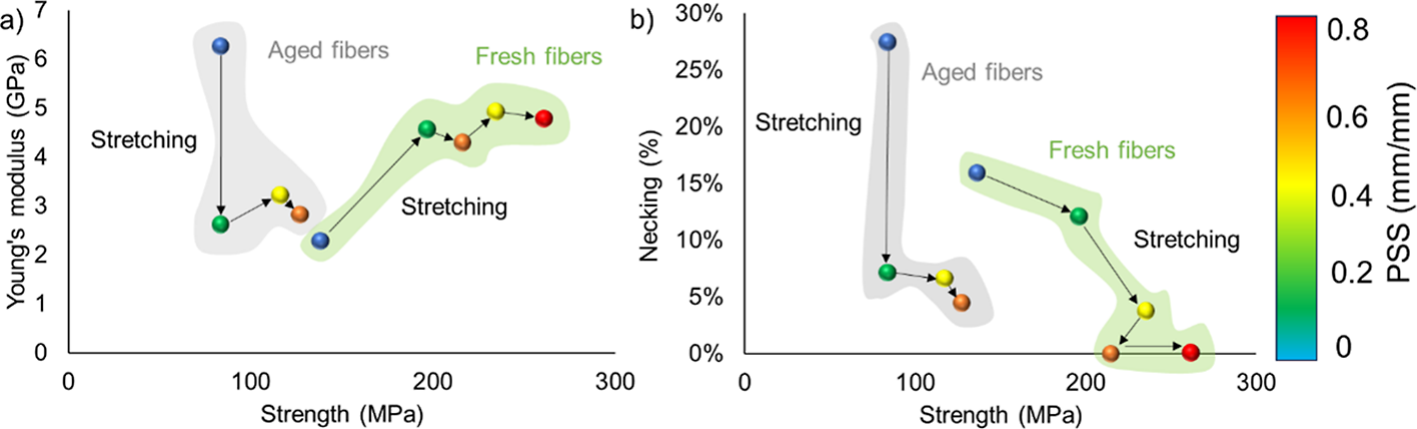
(a) Ashby plot of the Young’s modulus and strength of fresh and aged NT2RepCT artificial silk fibers that were PSS using Protocol I. (b) Ashby plot of the necking level and strength of fresh and aged NT2RepCT artificial silk fibers that underwent PSS using Protocol I. The graphs highlight the effects that aging and stretching have on fiber mechanical properties. The data from the fresh fibers were obtained from Schmuck et al.^6^

To summarize the effects of PSS and aging on our artificial silk fibers, we have created an Ashby plot containing the values of Young’s modulus and strength obtained from the artificial silk fibers described in Schmuck et al.^6^ and tested after exposure to different treatments (Figure 5). In general, aging increases the level of molecular disorder and makes the fiber Young’s modulus higher and fiber strength lower. To reverse these effects, PSS can be applied to increase the level of order in the protein chain network, and thereby increase the strength and Young’s modulus of the fibers.

## Conclusions

In this paper, we analyze the effects of PSS on the mechanical properties of wet-spun artificial silk fibers. We conclude that in addition to improving the strength and Young’s modulus of wet-spun fibers, PSS can restore the alignment of the polypeptide chains and partially revert the negative effects of aging. Finally, we propose that quantitative determination of necking can be used to assess polymeric fibers quality.

## Materials and Methods

### Spinning Artificial Spider Silk

The biomimetic artificial spider silk fibers were spun following an optimized protocol described in Schmuck et al.^6^ Briefly, the proteins (33 kDa in molecular weight, called NT2RepCT) were expressed in *E. coli* purified with immobilized metal ion chromatography in native conditions, also as previously described.^3^ To make the spinning dope, NT2RepCT stored in 20 mM Tris (pH 8) was concentrated to 300 mg/mL with an Amicon Ultra-15 centrifugal filter unit (Merck-Millipore) at 4000*g* and 4 °C with a 10 kDa cutoff membrane. The dope was then transferred to a 1 mL syringe which was connected to a pulled glass capillary having an orifice diameter of 42 ± 4 μm^54^. The spinning dope was then extruded at 17 μL/min into the spinning bath containing 4 L of a 750 mM acetate (Na) buffer at pH 5. The fibers were collected by a motored wheel spinning at 58 cm/s at the end of the 80 cm long spinning bath.

### Application of Post-spin Stretching and Tensile Tests

The application of a post-spin stretch was done in different ways (Protocols I and II) (Figure 2). The data for stretching fresh NT2RepCT fibers according to protocol I were obtained from Schmuck et al.^6^ and reanalyzed in this study. Briefly, in protocol I freshly spun fibers were mounted on paper frames with a 10 × 10 mm square window. Then, the samples were mounted on an 5943 tensile tester (Instron) and stretched in air at 6 mm/min up to different levels of strain (0.2, 0.4, 0.6, and 0.8, respectively). The fibers were allowed to rest at the desired level of strain for 10 min. Subsequently, they were removed from the tensile tester and remounted on new paper frames with a square window of 10 × 10 mm. The fibers were then allowed to rest for 1 day while mounted in the new paper frame. The diameter of the fibers was measured before and after the application of PSS, as described below. Finally, tensile tests on these fibers at 6 mm/min fibers were performed with the same Instron machine. Protocol I was also used for applying PSS to fibers that were aged for three months over the summer in the laboratory (humidity range 35–90% RH). In this case, however, PSS could not reach a strain level of 0.8 because fibers broke already around 0.6. For Protocol II, freshly spun fibers were mounted on paper frames with a 10 × 10 mm square window. Then, the samples were mounted on a Modular Stage Force (Linkam) device. These fibers were post-spin stretched in air up to different levels of strain (0.2, 0.4, 0.6, and 0.8, respectively) and allowed to rest for 10 min at the desired strain level. Then, without removing the fiber from the machine, the diameter was measured again, followed immediately by performing a tensile test on these fibers at 6 mm/min. Protocol II was used because it allowed the measurement of the diameter directly on the machine, which was not possible to do with Protocol I. The engineering stress was calculated by dividing the recorded load by the cross-sectional area (assumed to be circular) of the fibers. The engineering strain was calculated using the final (after post-spin stretching) gauge length, about 1 cm, and the measured displacement. Young’s modulus was obtained from the slope of the initial linear elastic part of the stress–strain curve. The toughness modulus was obtained by integrating the area under the stress–strain curves. Stress–strain curves were also recorded during PSS. All tensile tests, at least in ten replicates, were carried out at RH < 35% at room temperature.

### Measurement of the Fiber Diameters

The diameters of the fibers were measured at 5 different locations and then averaged. The diameters were measured before the tensile testing, employing light microscopy. For Protocol I, measurements were done using a Eclipse Ts2R-FL inverted microscope (Nikon) with a DFKNME33UX264 5 MP camera and a CFI Plan Fluor DL-10× objective. For Protocol II, the measurements were done as described previously using a Eclipse TE300 inverted microscope (Nikon) equipped with a DFK DFKNME33UX264 2.3 MP camera and a CFI Plan Fluor DL-10× objective.^53^ The fibers here analyzed did not show a uniform circular cross section. Thus, the average diameter was used to calculate the cross sectional area, assumed to be circular, which is a common practice in the silk field to obtain comparable results.^54^

### Measurement of the Birefringence Index

To measure birefringence index, we used a Microscope Axioscope 5/7 KMAT (Zeiss) equipped with a polarizer and a tilting Berek compensator (5λ). The birefringence index of the fibers was obtained by dividing the retardation of the polarized light by the thickness of the fiber (here represented by the diameter). This measurement was performed on the fibers that were subjected to PSS using protocol I.

## Supporting Information Available

All the stress strain curves of the fibers tested, as well as additional mechanical information (such as diameters and relaxation curves) are provided in the Supporting Information file (PDF)

## References

[ref1] Geyer R.; Jambeck J. R.; Law K. L. Production, Use, and Fate of All Plastics Ever Made. Sci. Adv. 2017, 3 (7), 25–29. 10.1126/sciadv.1700782. PMC5517107 28776036

[ref2] Andersson M.; Jia Q.; Abella A.; Lee X. Y.; Landreh M.; Purhonen P.; Hebert H.; Tenje M.; Robinson C. V.; Meng Q.; Plaza G. R.; Johansson J.; Rising A. Biomimetic Spinning of Artificial Spider Silk from a Chimeric Minispidroin. Nat. Chem. Biol. 2017, 13 (3), 262–264. 10.1038/nchembio.2269. 28068309

[ref3] Schmuck B.; Greco G.; Barth A.; Pugno N. M.; Johansson J.; Rising A. High-Yield Production of a Super-Soluble Miniature Spidroin for Biomimetic High-Performance Materials. Mater. Today 2021, 50, 16–23. 10.1016/j.mattod.2021.07.020.

[ref4] Arndt T.; Greco G.; Schmuck B.; Bunz J.; Shilkova O.; Francis J.; Pugno N. M.; Jaudzems K.; Barth A.; Johansson J.; Rising A. Engineered Spider Silk Proteins for Biomimetic Spinning of Fibers with Toughness Equal to Dragline Silks. Adv. Funct. Mater. 2022, 32, 2200986 10.1002/adfm.202200986. 36505976 PMC9720699

[ref5] Zhang D. Advances in Filament Yarn Spinning of Textiles and Polymers; The Textile Institute: Amsterdam, 2014; Vol. 21.

[ref6] Schmuck B.; Greco G.; Bäcklund F. G.; Pugno N. M.; Johansson J.; Rising A. Impact of Physio-Chemical Spinning Conditions on the Mechanical Properties of Biomimetic Spider Silk Fibers. Commun. Mater. 2022, 3 (1), 83 10.1038/s43246-022-00307-6.

[ref7] Nakajima T. Advanced Fiber Spinning Technology; Woodhead Publishing: Cambridge, UK, 1994; . 10.1533/9781845693213.

[ref8] Schmuck B.; Greco G.; Pessatti T. B.; Sonavane S.; Langwallner V.; Arndt T.; Rising A. Strategies for Making High-Performance Artificial Spider Silk Fibers. Adv. Funct. Mater. 2023, 34 (35), 2305040 10.1002/adfm.202305040. 39355086 PMC11440630

[ref9] Séguéla R. On the Natural Draw Ratio of Semi-Crystalline Polymers: Review of the Mechanical, Physical and Molecular Aspects. Macromol. Mater. Eng. 2007, 292, 235–244. 10.1002/mame.200600389.

[ref10] Kong D. C.; Yang M. H.; Zhang X. S.; Du Z. C.; Fu Q.; Gao X. Q.; Gong J. W. Control of Polymer Properties by Entanglement: A Review. Macromol. Mater. Eng. 2021, 306 (12), 2100536 10.1002/mame.202100536.

[ref11] Schwartz B. J. Conjugated Polymers As Molecular Materials: How Chain Conformation and Film Morphology Influence Energy Transfer and Interchain Interactions. Annu. Rev. Phys. Chem. 2003, 54 (1), 141–172. 10.1146/annurev.physchem.54.011002.103811. 12524429

[ref12] Xiangyang L.; Guanqun G.; Dong L.; Ye G.; Gu Y. Correlation between Hydrogen-Bonding Interaction and Mechanical Properties of Polyimide Fibers. Polym. Adv. Technol. 2009, 10 (4), 362–366. 10.1002/pat.1232.

[ref13] Turner M. J.; Thomas S. P.; Shi M. W.; Jayatilaka D.; Spackman M. A. Energy Frameworks: Insights into Interaction Anisotropy and the Mechanical Properties of Molecular Crystals. Chem. Commun. 2015, 51 (18), 3735–3738. 10.1039/C4CC09074H. 25525647

[ref14] He W.; Qian D.; Wang Y.; Zhang G.; Cheng Y.; Hu X.; Wen K.; Wang M.; Liu Z.; Zhou X.; Zhu M. A Protein-Like Nanogel for Spinning Hierarchically Structured Artificial Spider Silk. Adv. Mater. 2022, 34 (27), 2201843 10.1002/adma.202201843. 35509216

[ref15] An B.; Hinman M. B.; Holland G. P.; Yarger J. L.; Lewis R. V. Inducing β-Sheets Formation in Synthetic Spider Silk Fibers by Aqueous Post-Spin Stretching. Biomacromolecules 2011, 12 (6), 2375–2381. 10.1021/bm200463e. 21574576 PMC3503542

[ref16] Albertson A. E.; Teulé F.; Weber W.; Yarger J. L.; Lewis R. V. Effects of Different Post-Spin Stretching Conditions on the Mechanical Properties of Synthetic Spider Silk Fibers. J. Mech. Behav. Biomed. Mater. 2014, 29, 225–234. 10.1016/j.jmbbm.2013.09.002. 24113297 PMC4068612

[ref17] Madurga R.; Gañán-Calvo A. M.; Plaza G. R.; Atienza J. M.; Guinea G. V.; Elices M.; López P. A.; Daza R.; González-Nieto D.; Pérez-Rigueiro J. Comparison of the Effects of Post-Spinning Drawing and Wet Stretching on Regenerated Silk Fibers Produced through Straining Flow Spinning. Polymer (Guildf) 2018, 150, 311–317. 10.1016/j.polymer.2018.07.042.

[ref18] Asakura T.; Matsuda H.; Naito A.; Abe Y. Formylation of Recombinant Spider Silk in Formic Acid and Wet Spinning Studied Using Nuclear Magnetic Resonance and Infrared Spectroscopies. ACS Biomater. Sci. Eng. 2022, 8, 2390–2402. 10.1021/acsbiomaterials.2c00151. 35532754

[ref19] Asakura T.; Matsuda H.; Aoki A.; Naito A. Acetylation and Hydration Treatment of Recombinant Spider Silk Fiber, and Their Characterization Using 13C NMR Spectroscopy. Polymer (Guildf) 2022, 243 (January), 124605 10.1016/j.polymer.2022.124605.

[ref54] Greco G.; Schmuck B.; Jalali S. K.; Pugno N. M.; Rising A. Influence of Experimental Methods on the Mechanical Properties of Silk Fibers: A Systematic Literature Review and Future Road Map. Biophys. Rev. 2023, 4 (3), 031301 10.1063/5.0155552. PMC10903380 38510706

[ref20] Minguez R.; Barrenetxea L.; Solaberrieta E.; Lizundia E. A Simple Approach to Understand the Physical Aging in Polymers. Eur. J. Phys. 2019, 40 (1), 015502 10.1088/1361-6404/aaf244.

[ref21] Keten S.; Buehler M. J. Nanostructure and Molecular Mechanics of Spider Dragline Silk Protein Assemblies. J. R. Soc. Interface 2010, 7 (53), 1709–1721. 10.1098/rsif.2010.0149. 20519206 PMC2988266

[ref22] Buehler M. J. A Computational Building Block Approach towards Multiscale Architected Materials Analysis and Design with Application to Hierarchical Metal Metamaterials. Model. Simul. Mater. Sci. Eng. 2023, 31 (5), 054001 10.1088/1361-651X/accfb5.

[ref23] Lew A. J.; Jin K.; Buehler M. J. Designing Architected Materials for Mechanical Compression via Simulation, Deep Learning, and Experimentation. npj Comput. Mater. 2023, 9 (1), 80 10.1038/s41524-023-01036-1.

[ref24] G’sell C.; Aly-Helal N. A.; Jonas J. J. Effect of Stress Triaxiality on Neck Propagation during the Tensile Stretching of Solid Polymers. J. Mater. Sci. 1983, 18 (6), 1731–1742. 10.1007/BF00542069.

[ref25] Vincent P. I. The Necking and Cold-Drawing of Rigid Plastics. Polymer (Guildf) 1960, 1 (C), 7–19. 10.1016/0032-3861(60)90003-3.

[ref26] Zhang S.; Cao Z.; Gu X.; Ge T. Polymer Thin Film Necking: Ductility from Entanglements and Plane Stress Condition. Macromolecules 2024, 57 (13), 6221–6232. 10.1021/acs.macromol.4c00656.

[ref27] Greco G.; Mirbaha H.; Schmuck B.; Rising A.; Pugno N. M. Artificial and Natural Silk Materials Have High Mechanical Property Variability Regardless of Sample Size. Sci. Rep. 2022, 12 (1), 3507–3509. 10.1038/s41598-022-07212-5. 35241705 PMC8894418

[ref28] Heidebrecht A.; Eisoldt L.; Diehl J.; Schmidt A.; Geffers M.; Lang G.; Scheibel T. Biomimetic Fibers Made of Recombinant Spidroins with the Same Toughness as Natural Spider Silk. Adv. Mater. 2015, 27 (13), 2189–2194. 10.1002/adma.201404234. 25689835

[ref29] Zhang J.; Gong M.; Meng Q. Wet Spinning Is Employed to Produce Spider Silk with High Elasticity. APL Mater. 2023, 11 (8), 081110 10.1063/5.0160351.

[ref30] Nakamura H.; Kono N.; Mori M.; Masunaga H.; Numata K.; Arakawa K. Composition of Minor Ampullate Silk Makes Its Properties Different from Those of Major Ampullate Silk. Biomacromolecules 2023, 24 (5), 2042–2051. 10.1021/acs.biomac.2c01474. 37002945

[ref31] Sun M.; Zhang Y.; Zhao Y.; Shao H.; Hu X. The Structure-Property Relationships of Artificial Silk Fabricated by Dry-Spinning Process. J. Mater. Chem. 2012, 22 (35), 18372–18379. 10.1039/c2jm32576d.

[ref32] Plaza G. R.; Corsini P.; Pérez-Rigueiro J.; Marsano E.; Guinea G. V.; Elices M. Effect of Water on Bombyx Mori Regenerated Silk Fibers and Its Application in Modifying Their Mechanical Properties. J. Polym. Sci. 2008, 109, 1793–1801. 10.1002/app.28288.

[ref33] Pérez-rigueiro J.; Madurga R.; Gañán-calvo A. M.; Elices M.; Tasei Y.; Nishimura A.; Matsuda H. Emergence of Supercontraction in Regenerated Silkworm (Bombyx Mori) Silk Fibers. Sci. Rep. 2019, 9 (2398), 1–14. 10.1038/s41598-019-38712-6. 30787337 PMC6382804

[ref34] Greco G.; Schmuck B.; Del Bianco L.; Spizzo F.; Fambri L.; Pugno N. M.; Veintemillas-Verdaguer S.; Morales M. P.; Rising A. High-Performance Magnetic Artificial Silk Fibers Produced by a Scalable and Eco-Friendly Production Method. Adv. Compos. Hybrid Mater. 2024, 7 (5), 163 10.1007/s42114-024-00962-y. 39371407 PMC11447077

[ref35] Chen Y.; Han L.; Ju D.; Liu T.; Dong L. Disentanglement induced by uniaxial pre-stretching as a key factor for toughening poly(-lactic acid) sheets. Polymer (Guildf) 2018, 140, 47–55. 10.1016/j.polymer.2018.02.032.

[ref36] Nova A.; Keten S.; Pugno N. M.; Redaelli A.; Buehler M. J. Molecular and Nanostructural Mechanisms of Deformation, Strength and Toughness of Spider Silk Fibrils. Nano Lett. 2010, 10 (7), 2626–2634. 10.1021/nl101341w. 20518518

[ref37] He W.; Qian D.; Wang Y.; Zhang G.; Cheng Y.; Hu X.; Wen K.; Wang M.; Liu Z.; Zhou X.; Zhu M. A Protein-Like Nanogel for Spinning Hierarchically Structured Artificial Spider Silk. Adv. Mater. 2022, 34, 2201843 10.1002/adma.202201843. 35509216

[ref38] Meng F.; Pritchard R. H.; Terentjev E. M. Stress Relaxation, Dynamics, and Plasticity of Transient Polymer Networks. Macromolecules 2016, 49 (7), 2843–2852. 10.1021/acs.macromol.5b02667.

[ref39] Lamont S. C.; Mulderrig J.; Bouklas N.; Vernerey F. J. Rate-Dependent Damage Mechanics of Polymer Networks with Reversible Bonds. Macromolecules 2021, 54 (23), 10801–10813. 10.1021/acs.macromol.1c01943.

[ref40] Zhao P. C.; Li W.; Huang W.; Li C. H. A Self-Healing Polymer with Fast Elastic Recovery upon Stretching. Molecules 2020, 25 (3), 597 10.3390/molecules25030597. 32019143 PMC7037885

[ref41] Cohen N.; Levin M.; Eisenbach C. D. On the Origin of Supercontraction in Spider Silk. Biomacromolecules 2021, 22 (2), 993–1000. 10.1021/acs.biomac.0c01747. 33481568

[ref42] Semenov A. N.; Rubinstein M. Dynamics of Entangled Associating Polymers with Large Aggregates. Macromolecules 2002, 35 (12), 4821–4837. 10.1021/ma0117965.

[ref43] Keten S.; Xu Z.; Ihle B.; Buehler M. J. Nanoconfinement controls stiffness, strength and mechanical toughness of β-sheet crystals in silk. Nat. Mater. 2010, 9 (4), 359–367. 10.1038/nmat2704. 20228820

[ref44] Han Z.; Hao J.; Du C.; Yan H.; Yuan H.; Li K.; Wang L.; Xu Z.; Tan Y. Superior Strong and Stiff Alginate Fibers by Entanglement- Enhanced Stretching. Macromolecules 2023, 56, 6305–6315. 10.1021/acs.macromol.3c00380.

[ref45] Chen Y.-X.; Cai X.-Q.; Zhang G.-J. Topological Catenation Enhances Elastic Modulus of Single Linear Polycatenane. Chin. J. Polym. Sci. 2023, 41, 1486–1496. 10.1007/s10118-023-2902-x.

[ref46] Lepore E.; Isaia M.; Mammola S.; Pugno N. The Effect of Ageing on the Mechanical Properties of the Silk of the Bridge Spider Larinioides Cornutus (Clerck, 1757). Sci. Rep. 2016, 6 (1), 24699 10.1038/srep24699. 27156712 PMC4860589

[ref47] Brinson L. C.; Gates T. S. Effects of Physical Aging on Long Term Creep of Polymers and Polymer Matrix Composites. Int. J. Solids Struct. 1995, 32 (6–7), 827–846. 10.1016/0020-7683(94)00163-Q.

[ref48] Frieberg B. R.; Glynos E.; Sakellariou G.; Tyagi M.; Green P. F. Effect of Molecular Stiffness on the Physical Aging of Polymers. Macromolecules 2020, 53 (18), 7684–7690. 10.1021/acs.macromol.0c01331.

[ref49] Tu S.; Ren X.; He J.; Zhang Z. Stress–strain curves of metallic materials and post-necking strain hardening characterization: A review. Fatigue Fract. Eng. Mater. Struct. 2020, 43, 3–19. 10.1111/ffe.13134.

[ref50] Ward I. M. Mechanical Properties of Solid Polymers; Wiley Interscience: New York, 1980.

[ref51] Sun J.; He H.; Zhao K.; Cheng W.; Li Y.; Zhang P.; Wan S.; Liu Y.; Wang M.; Li M.; Wei Z.; Li B.; Zhang Y.; Li C.; Sun Y.; Shen J.; Li J.; Wang F.; Ma C.; Tian Y.; Su J.; Chen D.; Fan C.; Zhang H.; Liu K. Protein Fibers with Self-Recoverable Mechanical Properties via Dynamic Imine Chemistry. Nat. Commun. 2023, 14 (1), 5348 10.1038/s41467-023-41084-1. 37660126 PMC10475138

[ref52] Greco G.; Francis J.; Arndt T.; Schmuck B.; G Bäcklund F.; Barth A.; Johansson J.; M Pugno N.; Rising A. Properties of Biomimetic Artificial Spider Silk Fibers Tuned by PostSpin Bath Incubation. Molecules 2020, 25, 3248 10.3390/molecules25143248. 32708777 PMC7397010

[ref53] Bäcklund F. G.; Schmuck B.; Miranda G. H. B.; Greco G.; Pugno N. M.; Rydén J.; Rising A. An Image-Analysis-Based Method for the Prediction of Recombinant Protein Fiber Tensile Strength. Materials (Basel) 2022, 15 (3), 708 10.3390/ma15030708. 35160653 PMC8915176

[ref55] Xia X.-X.; Qian Z.-G.; Ki C. S.; Park Y. H.; Kaplan D. L.; Lee S. Y. Native-Sized Recombinant Spider Silk Protein Produced in Metabolically Engineered Escherichia Coli Results in a Strong Fiber. Proc. Natl. Acad. Sci. U.S.A. 2010, 107 (32), 14059–14063. 10.1073/pnas.1003366107. 20660779 PMC2922564

[ref56] Lin Z.; Deng Q.; Liu X. Y.; Yang D. Engineered Large Spider Eggcase Silk Protein for Strong Artificial Fibers. Adv. Mater. 2013, 25 (8), 1216–1220. 10.1002/adma.201204357. 23172740

[ref57] Copeland C. G.; Bell B. E.; Christensen C. D.; Lewis R. V. Development of a Process for the Spinning of Synthetic Spider Silk. ACS Biomater. Sci. Eng. 2015, 1 (7), 577–584. 10.1021/acsbiomaterials.5b00092. 27064312 PMC4826064

[ref58] Jones J. A.; Harris T. I.; Tucker C. L.; Berg K. R.; Christy S. Y.; Day B. A.; Gaztambide D. A.; Needham N. J. C.; Ruben A. L.; Oliveira P. F.; Decker R. E.; Lewis R. V. More Than Just Fibers: An Aqueous Method for the Production of Innovative Recombinant Spider Silk Protein Materials. Biomacromolecules 2015, 16 (4), 1418–1425. 10.1021/acs.biomac.5b00226. 25789668

[ref59] Peng Q.; Zhang Y.; Lu L.; Shao H.; Qin K.; Hu X.; Xia X. Recombinant Spider Silk from Aqueous Solutions via a Bio-Inspired Microfluidic Chip. Sci. Rep. 2016, 6, 36473 10.1038/srep36473. 27819339 PMC5098227

[ref60] Weatherbee-Martin N.; Xu L.; Hupe A.; Kreplak L.; Fudge D. S.; Liu X. Q.; Rainey J. K. Identification of Wet-Spinning and Post-Spin Stretching Methods Amenable to Recombinant Spider Aciniform Silk. Biomacromolecules 2016, 17 (8), 2737–2746. 10.1021/acs.biomac.6b00857. 27387592 PMC5770202

[ref61] Madurga R.; Gañán-Calvo A. M.; Plaza G. R.; Guinea G. V.; Elices M.; Pérez-Rigueiro J. Production of High Performance Bioinspired Silk Fibers by Straining Flow Spinning. Biomacromolecules 2017, 18 (4), 1127–1133. 10.1021/acs.biomac.6b01757. 28226209

[ref62] Wang J.; Fan T.; Hu X.; Huang W. Artificial Superstrong Silkworm Silk Surpasses Natural Spider Silks Artificial Superstrong Silkworm Silk Surpasses Natural Spider Silks. Matter 2022, 5 (7), 1–11. 10.1016/j.matt.2022.08.028.

[ref63] Thamm C.; Scheibel T. Recombinant Production, Characterization, and Fiber Spinning of an Engineered Short Major Ampullate Spidroin (MaSp1s). Biomacromolecules 2017, 18 (4), 1365–1372. 10.1021/acs.biomac.7b00090. 28233980

[ref64] Xu L.; Lefèvre T.; Orrell K. E.; Meng Q.; Auger M.; Liu X. Q.; Rainey J. K. Structural and Mechanical Roles for the C-Terminal Nonrepetitive Domain Become Apparent in Recombinant Spider Aciniform Silk. Biomacromolecules 2017, 18 (11), 3678–3686. 10.1021/acs.biomac.7b01057. 28934550 PMC5762186

[ref65] Kamada A.; Mittal N.; Söderberg L. D.; Ingverud T.; Ohm W.; Roth S. V.; Lundell F.; Lendel C. Flow-Assisted Assembly of Nanostructured Protein Microfibers. Proc. Natl. Acad. Sci. U.S.A. 2017, 114 (6), 1232–1237. 10.1073/pnas.1617260114. 28123065 PMC5307460

[ref66] Bowen C. H.; Dai B.; Sargent C. J.; Bai W.; Ladiwala P.; Feng H.; Huang W.; Kaplan D. L.; Galazka J. M.; Zhang F. Recombinant Spidroins Fully Replicate Primary Mechanical Properties of Natural Spider Silk. Biomacromolecules 2018, 19, 3853–3860. 10.1021/acs.biomac.8b00980. 30080972

[ref67] Xu L.; Weatherbee-Martin N.; Liu X. Q.; Rainey J. K. Recombinant Silk Fiber Properties Correlate to Prefibrillar Self-Assembly. Small 2019, 15 (12), 1–12. 10.1002/smll.201805294. 30756524

[ref68] Gonska N.; López P. A.; Lozano-Picazo P.; Thorpe M.; Guinea G. V.; Johansson J.; Barth A.; Pérez-Rigueiro J.; Rising A. Structure-Function Relationship of Artificial Spider Silk Fibers Produced by Straining Flow Spinning. Biomacromolecules 2020, 21 (6), 2116–2124. 10.1021/acs.biomac.0c00100. 32223220

[ref69] Wen R.; Wang K.; Meng Q. Characterization of the Second Type of Aciniform Spidroin (AcSp2) Provides New Insight into Design for Spidroin-Based Biomaterials. Acta Biomater. 2020, 115, 210–219. 10.1016/j.actbio.2020.08.009. 32798722

[ref70] Zhang C.; Mi J.; Qi H.; Huang J.; Liu S.; Zhang L.; Fan D. Engineered a Novel PH-Sensitive Short Major Ampullate Spidroin. Int. J. Biol. Macromol. 2020, 154, 698–705. 10.1016/j.ijbiomac.2020.03.153. 32198037

[ref71] Zhu H.; Sun Y.; Yi T.; Wang S.; Mi J.; Meng Q. Tough Synthetic Spider-Silk Fibers Obtained by Titanium Dioxide Incorporation and Formaldehyde Cross-Linking in a Simple Wet-Spinning Process. Biochimie 2020, 175, 77–84. 10.1016/j.biochi.2020.05.003. 32417459

[ref72] Li X.; Mi J.; Wen R.; Zhang J.; Cai Y.; Meng Q.; Lin Y. Wet-Spinning Synthetic Fibers from Aggregate Glue: Aggregate Spidroin 1 (AgSp1). ACS Appl. Bio Mater. 2020, 3 (9), 5957–5965. 10.1021/acsabm.0c00619. 35021824

[ref73] Li J.; Zhu Y.; Yu H.; Dai B.; Jun Y. S.; Zhang F. Microbially Synthesized Polymeric Amyloid Fiber Promotes β-Nanocrystal Formation and Displays Gigapascal Tensile Strength. ACS Nano 2021, 15 (7), 11843–11853. 10.1021/acsnano.1c02944. 34251182

[ref74] Fan T.; Qin R.; Zhang Y.; Wang J.; Fan J. S.; Bai X.; Yuan W.; Huang W.; Shi S.; Su X. C.; Yang D.; Lin Z. Critical Role of Minor Eggcase Silk Component in Promoting Spidroin Chain Alignment and Strong Fiber Formation. Proc. Natl. Acad. Sci. U.S.A. 2021, 118 (38), e2100496118 10.1073/pnas.2100496118. 34531321 PMC8463894

[ref75] Li X.; Qi X.; Cai Y. M.; Sun Y.; Wen R.; Zhang R.; Johansson J.; Meng Q.; Chen G. Customized Flagelliform Spidroins Form Spider Silk-like Fibers at PH 8.0 with Outstanding Tensile Strength. ACS Biomater. Sci. Eng. 2022, 8 (1), 119–127. 10.1021/acsbiomaterials.1c01354. 34908395 PMC8753598

[ref76] Jin Q.; Pan F.; Hu C. F.; Lee S. Y.; Xia X. X.; Qian Z. G. Secretory Production of Spider Silk Proteins in Metabolically Engineered Corynebacterium Glutamicum for Spinning into Tough Fibers. Metab. Eng. 2022, 70 (January), 102–114. 10.1016/j.ymben.2022.01.009. 35065259

[ref77] Ye X.; Capezza A. J.; Davoodi S.; Wei X.; Andersson R. L.; Chumakov A.; Roth S. V.; Langton M.; Lundell F.; Hedenqvist M. S.; et al. Robust Assembly of Cross-Linked Protein Nanofibrils into Hierarchically Structured Microfibers. ACS Nano 2022, 16, 12471–12479. 10.1021/acsnano.2c03790. 35904348 PMC9413408

[ref78] Yao J.; Chen S.; Chen Y.; Wang B.; Pei Q.; Wang H. Macrofibers with High Mechanical Performance Based on Aligned Bacterial Cellulose Nanofibers. ACS Appl. Mater. Interfaces 2017, 9 (24), 20330–20339. 10.1021/acsami.6b14650. 28045246

[ref79] Greco G.; Arndt T.; Schmuck B.; Francis J.; Bäcklund F. G.; Shilkova O.; Barth A.; Gonska N.; Seisenbaeva G.; Kessler V.; Johansson J.; Pugno N. M.; Rising A. Tyrosine Residues Mediate Supercontraction in Biomimetic Spider Silk. Commun. Mater. 2021, 2 (1), 43 10.1038/s43246-021-00147-w.

[ref80] Cheng J.; Hu C.; Gan C.; Xia X.; Qian Z. Functionalization and Reinforcement of Recombinant Spider Dragline Silk Fibers by Confined Nanoparticle Formation. ACS Biomater. Sci. Eng. 2022, 8, 3299–3309. 10.1021/acsbiomaterials.2c00209. 35820196

