# Supplementary material for: Post-spin Stretch Improves Mechanical Properties, Reduces Necking, and Reverts Effects of Aging in Biomimetic Artificial Spider Silk Fibers
Source: ACS Appl Polym Mater. 2024 Nov 20;6(23):14342–50. doi: 10.1021/acsapm.4c02192 (PMC11650584; doi:10.1021/acsapm.4c02192)
Supplement: Supplementary file 1 — ap4c02192_si_001.pdf [file ap4c02192_si_001.pdf]

Supporting information

Post-spin stretch improves mechanical properties, reduces necking, and reverts effects of aging in biomimetic artificial spider silk fibers

Gabriele Greco[1]*, Benjamin Schmuck[1,2], Fredrik G. Bäcklund[3], Günter Reiter[4] and Anna Rising[1,2]*

[1] Department of Anatomy, Physiology and Biochemistry, Swedish University of Agricultural Sciences, Box 7011, 750 07 Uppsala, Sweden

[2] Department of Medicine Huddinge, Karolinska Institutet, 141 83 Huddinge, Sweden

[3] RISE Research Institutes of Sweden, Division Materials and Production, Department of Polymers, Fibers and Composites, 431 53 Mölndal, Sweden Xxx Please add your affiliation

[4] Physikalisches Institut, Albert-Ludwigs-Universität Freiburg, Hermann-Herder-Straße 3, Freiburg 79104, Germany

* corresponding author: gabriele.greco@slu.se

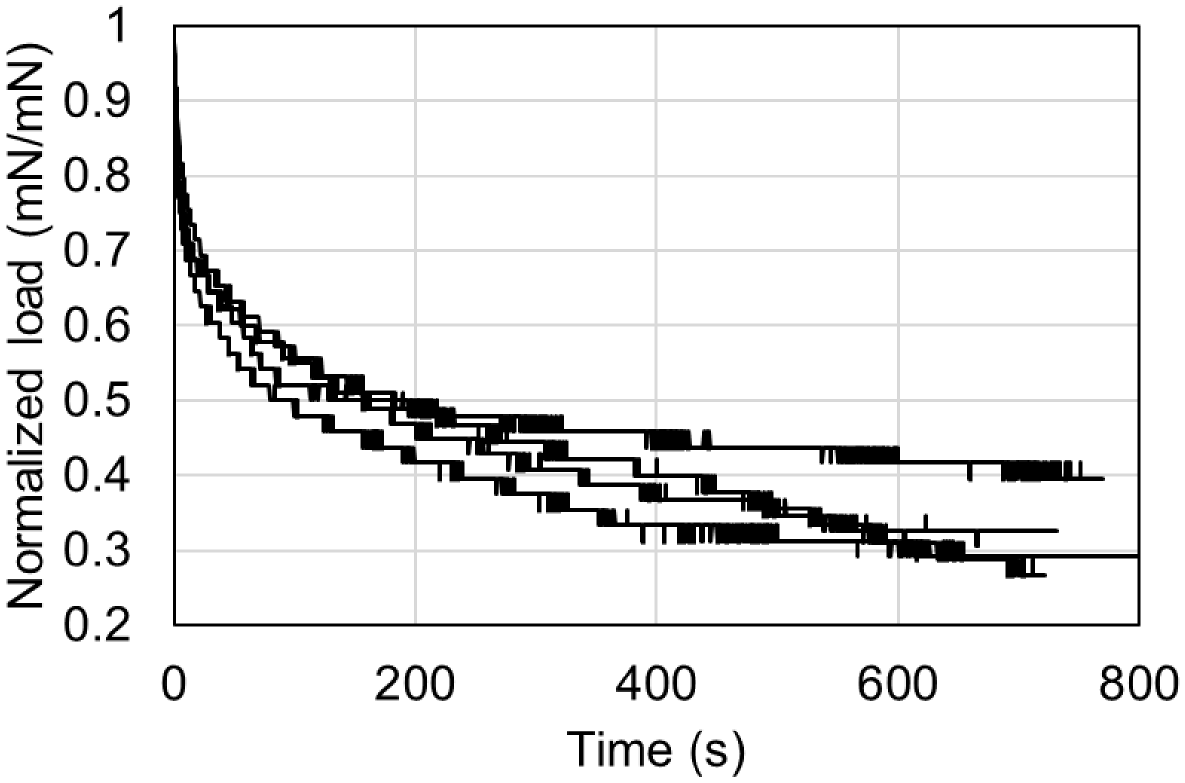

Figure S1: Representative relaxation curves of the artificial spider silk fibers made from NT2RepCT, indicating that the majority of residual stress dissipates within 10 minutes.

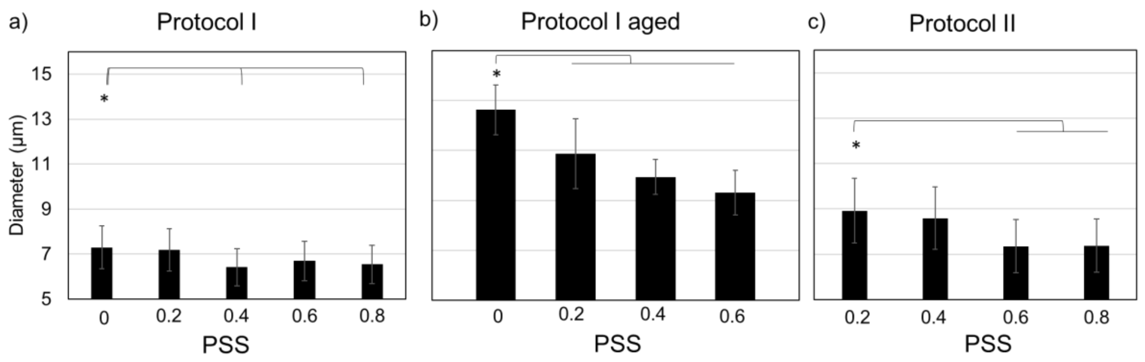

Figure S2: Diameter of the artificial spider silk fibers made from NT2RepCT plotted vs the applied post-spin stretching for a) Protocol I, b) Protocol I applied to aged fibers, and c) Protocol II. The Data of the panels a) were obtained from Schmuck et al.[6]. Stars indicate that the difference is significant with p-value<0.05 and the error bars are the standard deviations.

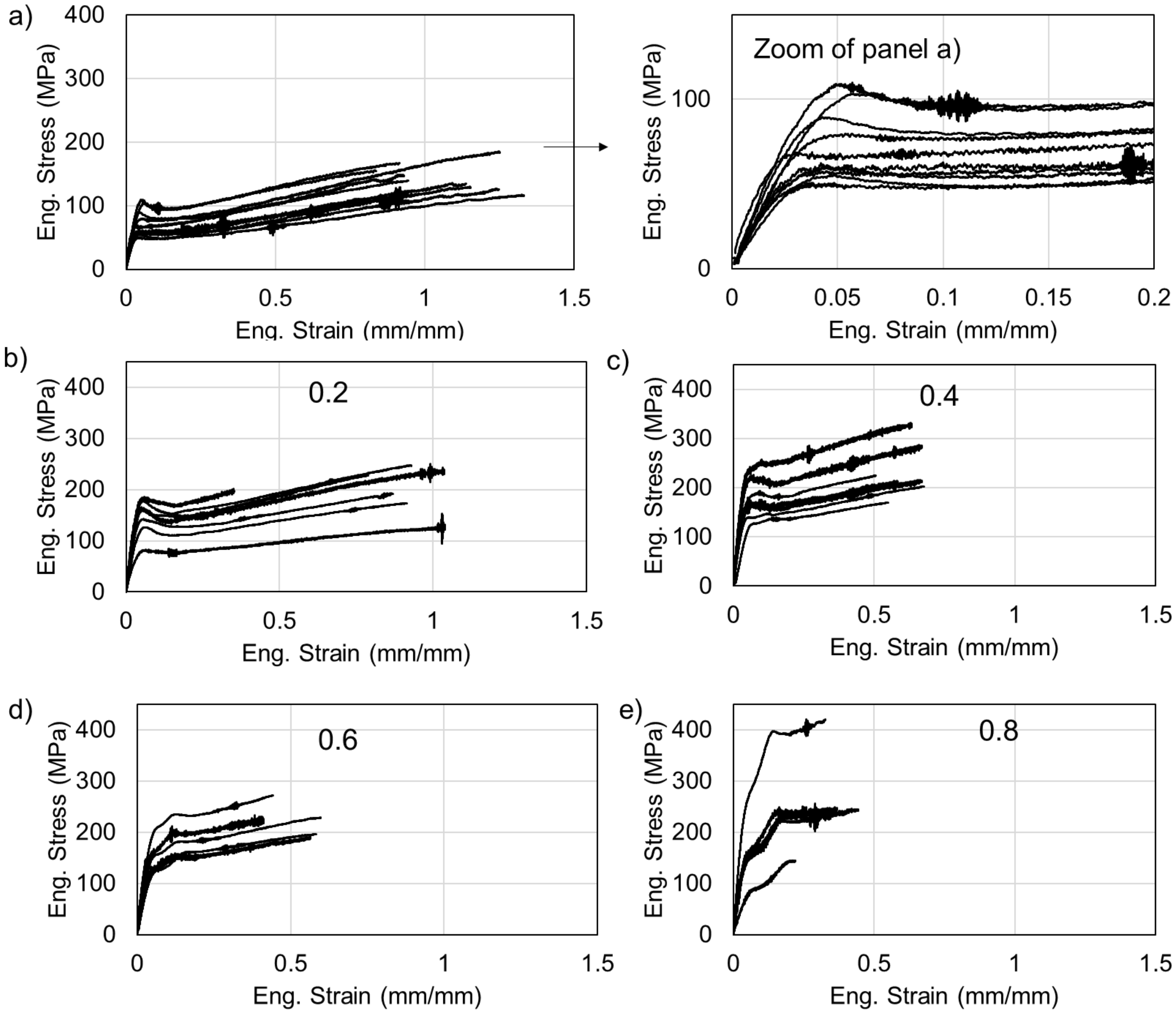

Figure S3: Representative stress-strain curves of the artificial spider silk fibers made from NT2RepCT a) in the control state (with a side panel which is the zoom of the necking region) and b-e) with different levels of post-spin stretching applied with Protocol I. These Data were obtained from Schmuck et al.[7].

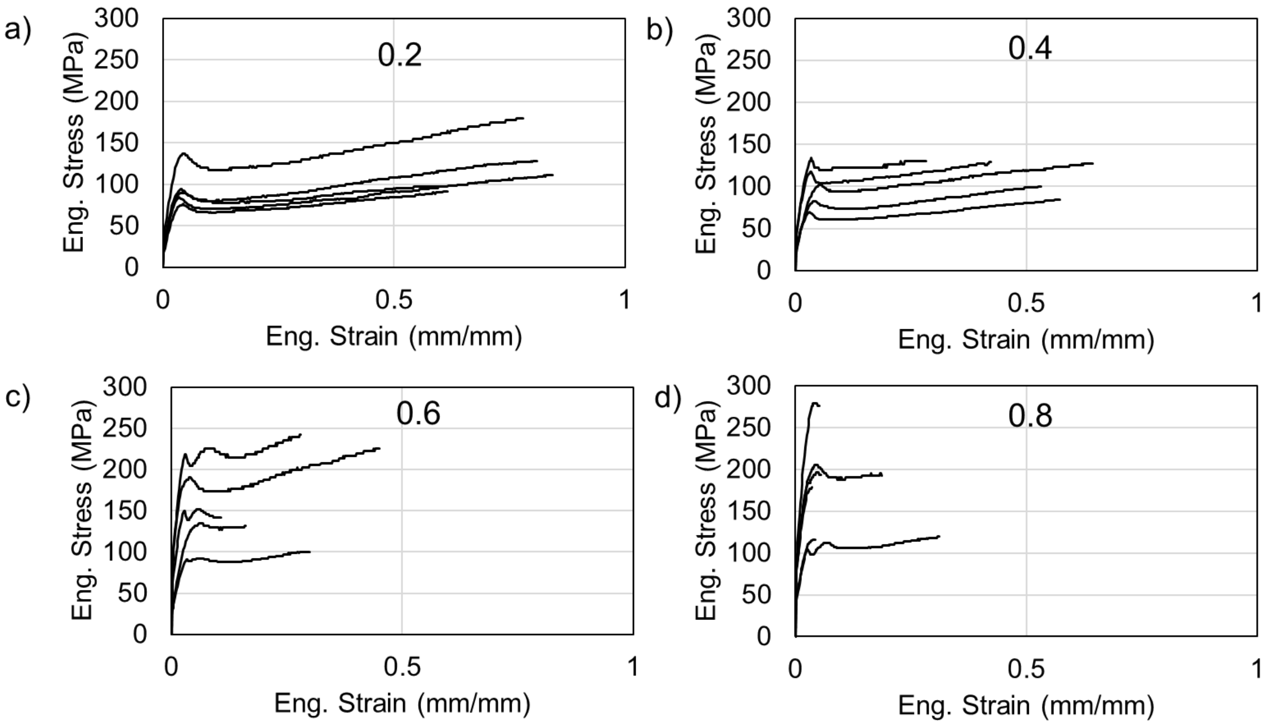

Figure S4: Representative stress-strain curves of the artificial spider silk fibers made from NT2RepCT in the a-e) with different levels of post-spin stretching applied with Protocol II.

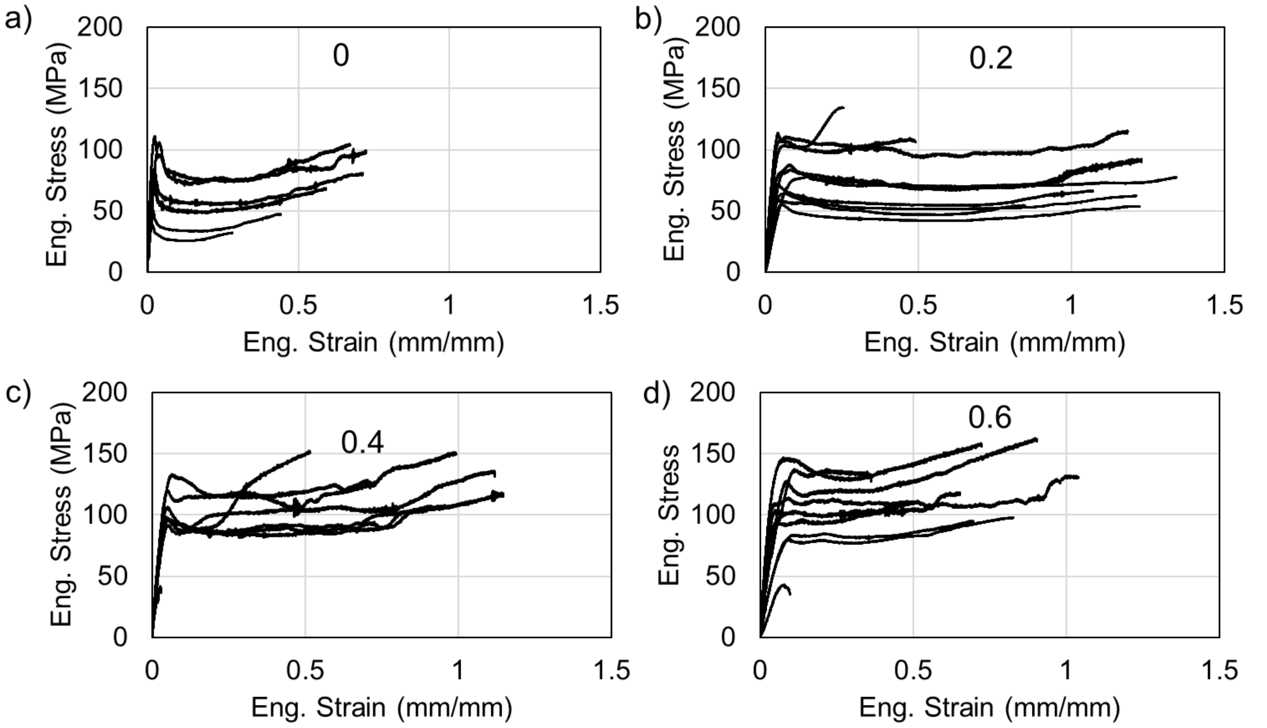

Figure S5: Representative stress-strain curves of the artificial spider silk fibers made from NT2RepCT in the a) control stated and b-d) with different levels of post-spin stretching applied with Protocol I, after being aged for 3 months.
